# ﻿Two new thomisid species (Arachnida, Araneae, Thomisidae) from China and Vietnam, with the first descriptions of the males of *Borboropactuslongidens* Tang & Li, 2010 and *Stephanopisxiangzhouica* Liu, 2022

**DOI:** 10.3897/zookeys.1159.102601

**Published:** 2023-05-02

**Authors:** Cong-zheng Li, Yan-bin Yao, Yong-hong Xiao, Ke-ke Liu

**Affiliations:** 1 College of Life Science, Jinggangshan University, Ji’an 343009, Jiangxi, China Jinggangshan University Ji’an China; 2 Jinshan College of Fujian Agriculture and Forestry University, Fuzhou 350007, Fujian, China Jinshan College of Fujian Agriculture and Forestry University Fuzhou China

**Keywords:** Crab spider, distribution, new species, single sex, taxonomy

## Abstract

Collections of thomisid spiders by amateur and professional arachnologists in China have led to the discovery of some interesting crab spiders (Thomisidae). Two new species in two genera of thomisid spiders are described and illustrated with photographs and SEMs: *Phartaxizang* Liu & Yao, **sp. nov.** (♀) and *Stephanopisqiong* Liu & Yao, **sp. nov.** (♀). The previously unknown males of *Borboropactuslongidens* Tang & Li, 2010 and *Stephanopisxiangzhouica* Liu, 2022 were also collected and are described for the first time. The genus *Borboropactus* Simon, 1884 is reported for the first time from Vietnam. The new *Stephanopis* species is also recorded for only the second time from the Asian mainland. Distributions of all these species are mapped.

## ﻿Introduction

The family Thomisidae, the crab spiders, is the seventh largest spider family with a global distribution, comprising 2165 extant species belonging to 171 genera (WSC 2022). Of these, 312 species from 53 genera were recorded from China (WSC 2022). More than 50% of the Chinese species are from southern provinces of this country, such as Yunnan, Hainan and Hunan. Only a few percent of the total Chinese number of species have been reported in the past ten years (Li and Lin 2016; Liu et al. 2017, 2022b, 2022c; Yu and Zhang 2017; Yu et al. 2017; Tian et al. 2018; Lin et al. 2019, 2022; Huang and Lin 2020; Wang et al. 2020; Liu et al. 2021; Zhang et al. 2022). China not only has the most species- and genus-rich thomisid fauna, but for approximately 65%, only one of the sexes has been described, which represents a challenge for future taxonomic revisions (Li 2020). However, only a few papers (Meng et al. 2019; Wang et al. 2020; Liu et al. 2022c; Lin et al. 2022) have revised the species of these single-sex species, and there are still many poorly known species from southern China with unusual morphological characteristics (Liu et al. 2022a).

In the past five years, specimens have been collected by spider enthusiasts and colleagues. When examining these spider specimens collected from Tibet, Guangdong, Fujian and Hainan provinces, two new thomisid species were identified, two males of *Borboropactuslongidens* Tang & Li, 2010 and *Stephanopisxiangzhouica* Liu, 2022 were found for the first time. The aims of the present paper are (1) to provide detailed descriptions of two new species, (2) to provide descriptions of previously unknown males of these two species and (3) to provide the second record of the genus *Stephanopis* from the Asian mainland.

## ﻿Materials and methods

Specimens were examined using a SZ6100 stereomicroscope. Both male and female copulatory organs were dissected and examined in 95% ethanol using an Olympus CX43 compound microscope with a KUY NICE CCD camera. The epigynes were cleared with pancreatin solution (Álvarez-Padilla and Hormiga 2007). Specimens, including dissected male palps and epigynes, were preserved in 75% ethanol after examination. For SEM photographs, the specimens were dried under natural conditions and photographed with a ZEISS EVO LS15 scanning electron microscope. Specimens, including the detached male palps or female genitalia, were stored in 75% ethanol after examination. Types are deposited in the Animal Specimen Museum, College of Life Science, Jinggangshan University (**ASM-JGSU**).

All morphological measurements were taken using a Zeiss Stereo Discovery V12 stereomicroscope with Zoom Microscope System (Software: AxioVision SE64 Version 4.8.3) and are given in millimetres. The body length of all specimens was taken from the anterior margin of the clypeus to the posterior end of the abdomen, excluding the spinnerets. Terminology of the male and female genitalia follows Benjamin (2011), Meng et al. (2019), and Liu et al. (2022c). Leg measurements are given as total length (femur, patella, tibia, metatarsus, tarsus). Leg spines were documented by dividing each leg segment into four aspects: dorsal (d), prolateral (p), retrolateral (r) and ventral (v).

The abbreviations used in the figures and text are as follows:

**ALE** anterior lateral eye;

**AME** anterior median eye;

**At** atrium;

**CD** copulatory duct;

**CO** copulatory opening;

**Con** conductor;

**d** dorsal;

**Em** embolus;

**ET** epigynal tooth;

**FD** fertilization duct;

**GA** glandular appendage;

**IZCAS**Institute of Zoology, Chinese Academy of Sciences;

**MA** median apophysis;

**MF** median field;

**MOA** median ocular area;

**MS** membranous sac;

**p** prolateral;

**PLE** posterior lateral eye;

**PME** posterior median eye;

**r** retrolateral;

**RTA** retrolateral tibial apophysis;

**Sp** spermatheca;

**St** subtegulum;

**TR** transverse ridge of copulatory opening;

**v** ventral;

**VTA** ventral tibial apophysis.

## ﻿Taxonomy

**Family Thomisidae Sundevall, 1833**

### ﻿Genus Borboropactus

Taxon classificationAnimaliaAraneaeThomisidae

Simon, 1884

04EB53BB-E81F-5C90-A4E0-A2A1EF7BF2AB

#### Comments.

This genus includes 17 species, all of which are distributed in tropical Africa and Asia (WSC 2022). Most species (10 species) are described based either on single females or single males (Li and Lin 2016) and taxonomic species identification is therefore challenging. In China, two species are known only from a single sex, *B.biprocessus* Tang, Yin & Peng, 2012 (male) and *B.longidens* Tang & Li, 2010 (female) (Tang and Li 2010; Yin et al. 2012).

### ﻿ Borboropactus longidens

Taxon classificationAnimaliaAraneaeThomisidae

Tang & Li, 2010

204C8797-3379-5983-A4E9-D03825132EEB

Figs 1 , 2 , 3

Borboropactus longidens Tang & Li, 2010: 21, figs 15A−D, 16A, B (♀).

#### Material examined.

1 ♂, 1 ♀, China, Hainan, Ledong County, Jianfengling National Natural Reserve, Mingfenggu Scenic Spot, 18°44'25.87"N, 108°50'47.83"E, 1–31 May 2021, Yunhu Mo leg. (Tho-293, ASM-JGSU); 1 ♂, 2 ♀, Vietnam, Tam Dao National Park, Vinh Phuc, Vietnam Natural Forest, 21°29.55'N, 105°37.42'E, 1063 m, 12 September. 2007, Pham Dinh Sac leg. (IZCAS, examined by Yejie Lin).

#### Diagnosis.

**Figure 1.**
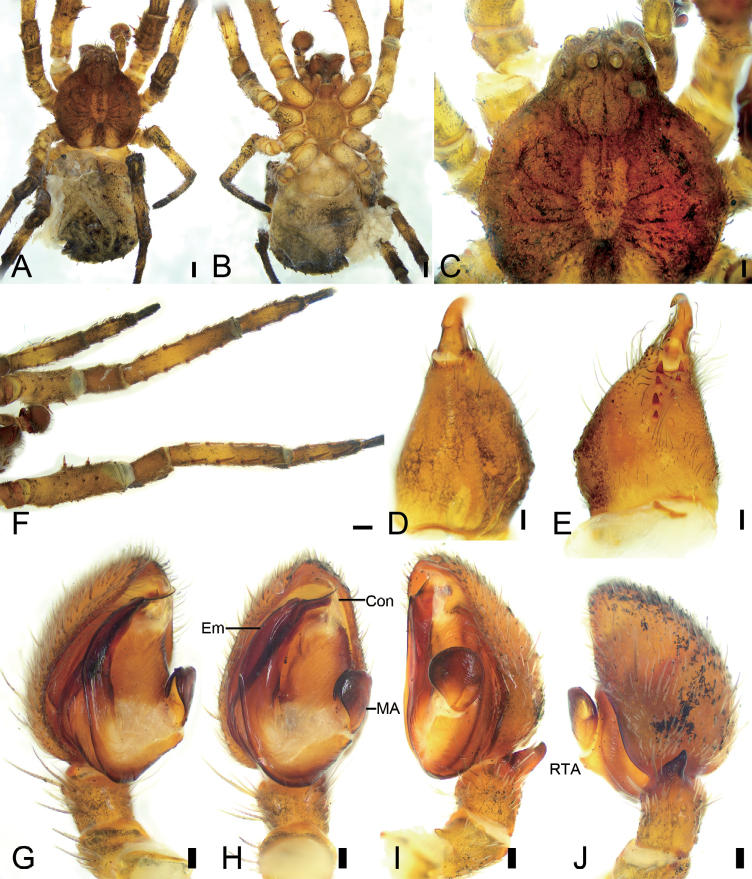
*Borboropactuslongidens* Tang & Li, 2010, male **A** habitus, dorsal view **B** same, ventral view **C** eyes, dorsal view **D** chelicera, dorsolateral view **E** same, ventral view **F** leg I, ventral view **G** palp, ventro-prolateral view **H** same, ventral view **I** same, ventro-retrolateral view **J** same, retro-dorsal view. Abbreviations: Con – conductor, Em – embolus, MA – median apophysis, RTA – retrolateral tibial apophysis. Scale bars: 0.5 mm (**A, B**); 0.2 mm (**C**); 0.1 mm (**D–J**).

**Figure 2.**
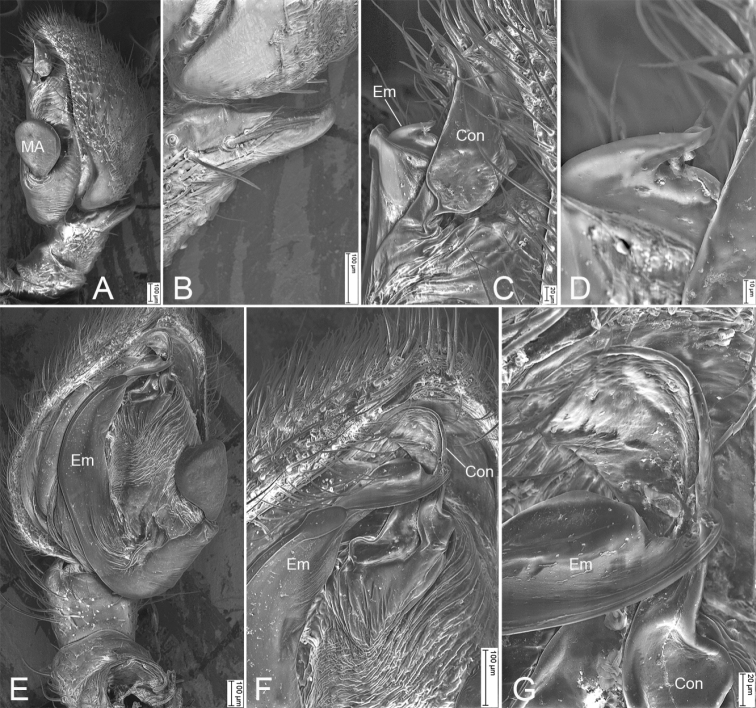
SEM micrographs of *Borboropactuslongidens* Tang & Li, 2010, male palp **A** retrolateral view **B** same, details of retrolateral tibial apophysis **C** same, details of conductor and embolus **D** same, details of embolic tip **E** ventral view **F** same, detail of conductor and embolus **G** same, details of conductor and embolic tip. Abbreviations: Con – conductor, Em – embolus, MA – median apophysis, RTA – retrolateral tibial apophysis.

**Figure 3.**
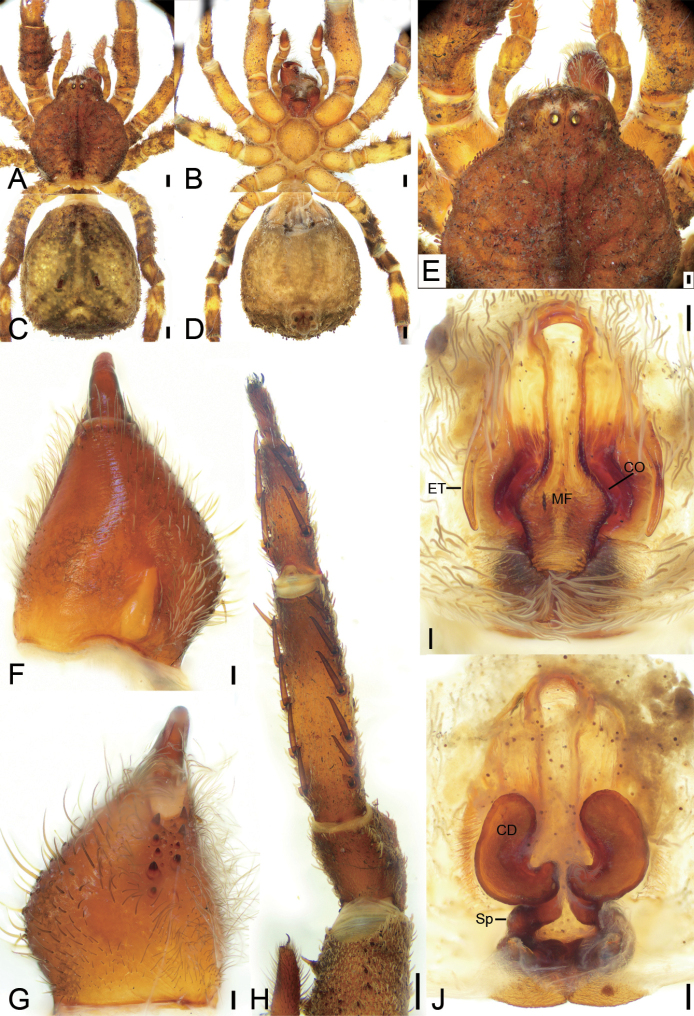
*Borboropactuslongidens* Tang & Li, 2010, female **A** prosoma, dorsal view **B** same, ventral view **C** opisthosoma, dorsal view **D** same, ventral view **E** eyes, dorsal view **F** chelicera, dorsal view **G** same, ventral view **H** leg I, ventral view **I** epigyne, dorsal view **J** same, ventral view. Abbreviations: CD – copulatory duct, CO – copulatory opening, ET – epigynal tooth, MF – median field, Sp – spermatheca. Scale bars: 0.5 mm (**A–D**); 0.2 (**E**); 0.1 mm (**F–J**).

The male of this species resembles that of *Borboropactusedentatus* Tang & Li, 2010 (see Tang and Li 2010: 12, fig. 6A−D) by having the embolus lacking the spiralling tip, but can be easily distinguished by the round median apophysis (vs. oval in *B.edentatus*), the tibia with a horn-like retrolateral apophysis as long as the tibia (vs. triangular, shorter than tibia in *B.edentatus*), and lacking the dorsal apophysis (vs. present in *B.edentatus*) (Figs 1G−J, 2). The female of this new species differs from that of *B.edentatus* (see Tang and Li 2010: 12, fig. 7B, C) by the narrow median field (vs. lacking), the slender epigynal teeth (vs. lacking), and the L-shaped copulatory ducts (vs. oval) (Fig. 3I, J).

#### Description.

**Male**. ***Habitus*** as in Fig. 1A, B. Total length 7.15, prosoma length 3.36, width 3.00, anteriorly narrowed to 0.41× its maximum width. Eye diameters (Fig. 1C): AME 0.16, ALE 0.17, PME 0.15, PLE 0.20; interdistances: AME−AME 0.12, AME−ALE 0.14, PME−PME 0.17, PME−PLE 0.31, AME−PME 0.21, AME−PLE 0.45, ALE−ALE 0.73, PLE−PLE 1.03, ALE−PLE 0.20. MOA 0.51 long, front width 0.43, back width 0.44. Chelicerae (Fig. 1D, E) with four promarginal teeth, three retromarginal teeth, including a vestige tooth, and four small denticles in-between the teeth. Endites (Fig. 1B) nearly quadrilateral, with dense setae on surface. Labium (Fig. 1B) rectangular, anteriorly with strong setae. Sternum (Fig. 1B) broadly oval, with dense setae around margin. Legs measurements: I 10.58 (3.1, 1.55, 3, 2.02, 0.91); II 7.45 (2.5, 0.88, 2.32, 1.19, 0.56); III 7.63 (1.88, 0.95, 2.11, 1.91, 0.78); IV 9.22 (2.01, 1.91, 2.01, 2.16, 1.13); spination (Fig. 1A, B, F): I Fe: p2, v2; Ti: d4, v10; Mt: d3, v6; II Pa: d1; Ti: d4, v10; Mt: d2, v6; III Fe: d1; Ti: d3; Mt: d3; IV: Fe: d2; Ti: d4; Mt: d3; cusps: I Fe: 8; II Fe: 1. Opisthosoma (Fig. 1A, B) length 3.79, width 3.22, dorsally with abundant macrosetae on posterior part.

***Colouration*** (Fig. 1A, B). Prosoma yellow to dark brown, densely covered white feathery setae, with an approximate U-shaped yellowish marking medially and dark thin radial markings around the fovea. Chelicerae, endites, and labium yellow-brown. Sternum yellow. Legs from yellow to dark brown, mottled. Opisthosoma yellow to greyish black.

***Palp*** (Figs 1G−J, 2). Palp with a relative long and strong retrolateral tibial apophysis (*RTA*), extending dorsally, as long as tibia in retrolateral view; median apophysis (*MA*) pear-shaped, located at submedian-retrolateral of tegulum; conductor (*Con*) translucent, with broad base and apex, nearly as long as 1/3 of tegulum; embolus (*Em*) flatted-shaped, slightly less than tegular length, originating at the 6 o’clock position of tegulum, with a membranous anterior part and spine-like apex.

**Female. *Habitus*** as in Fig. 3A−D. As in male except as follows. Total length 10.67, prosoma length 4.46, width 4.32, anteriorly narrowed to 0.44× its maximum width. Eye diameters (Fig. 3E): AME 0.16, ALE 0.19, PME 0.17, PLE 0.20; interdistances: AME−AME 0.15, AME−ALE 0.27, PME−PME 0.24, PME−PLE 0.47, AME−PME 0.34, AME−PLE 0.68, ALE−ALE 0.99, PLE−PLE 1.44, ALE−PLE 0.36. MOA 0.64 long, front width 0.45, back width 0.54. Chelicerae (Fig. 3F, G) with five promarginal teeth, four retromarginal teeth, including a vestige tooth, and nine small denticles in-between teeth. Labium (Fig. 3B) wider than long. Legs (Fig. 3A−D, H) measurements: I 10.91 (3.45, 1.35, 3.35, 2.01, 0.75); II 8.73 (2.5, 1.25, 2.41, 1.88, 0.69); III 9.95 (2.67, 1.11, 2.5, 2.53, 1.14); IV 10.3 (2.75, 1.52, 2.38, 2.44, 1.21); spination (Fig. 3A−D, H): I Fe: p2; Ti: v11; Mt: d3, v6; II Ti: d3, v9; Mt: d3, v6; III Fe: d1; Ti: d4; Mt: d3; cusps: I Fe: 18; II Fe: 1; IV Fe: 1. Opisthosoma (Fig. 3C, D) length 6.21, width 5.32.

***Colouration*** (Fig. 3A−D). Prosoma medially with a fine dark mark. Chelicerae, endites, and labium red-brown. Opisthosoma white to dark brown.

***Epigyne*** (Fig. 3I, J). Median field (*MF*) flask-like, subposterior part broader than other parts; epigynal teeth (*ET*) very long, as long as 1/2 of median field, arising median-bilaterally; copulatory openings (*CO*) arising from anterior part of maximum median field; copulatory ducts (*CD*) broad, wider than spermathecae, both ends swollen, sloping C-shaped, located at median of vulva, anterior part widely separated by its maximum width, and posterior part are approaching each other; spermathecae (*Sp*) C-shaped, median part have a constriction, posterior part close touching, both ends slightly swollen.

#### Distribution.

Known from China (Hainan) and Vietnam (Fig. 10).

### ﻿Genus Pharta

Taxon classificationAnimaliaAraneaeThomisidae

Thorell, 1891

9B7E2AD6-B92B-5A28-A4B2-A7E66CAF5CEC

#### Comments.

This genus includes ten species distributed in Asia (Benjamin 2014; WSC 2022). Half of them (5 species) are recorded from China, where it is known from Yunnan, Guizhou, and Jiangxi provinces (Tang et al. 2009; Wang et al. 2016; Liu et al. 2022c). No species were recorded from Tibet Province.

### ﻿ Pharta xizang

Taxon classificationAnimaliaAraneaeThomisidae

Liu & Yao sp. nov.

F8C560B9-4CDC-5227-94ED-5B448B79A772

https://zoobank.org/4B8E9C66-615E-4DCC-93A5-5A439C23ADB3

Fig. 4

#### Type material.

***Holotype*** ♀: China, Tibet, Linzhi City, Motuo County, near Lianhua Hotel, 29°19'31.07"N, 95°19'59.51"E, 1101 m, 17 July 2017, Jian Chen, Jie Liu, Man Fang, Zengtao Zhang and Fengxiang Liu leg. (Tho-296, ASM-JGSU).

#### Etymology.

The specific name derived from the Chinese Pinyin for Tibet; noun in apposition.

#### Diagnosis.

**Figure 4.**
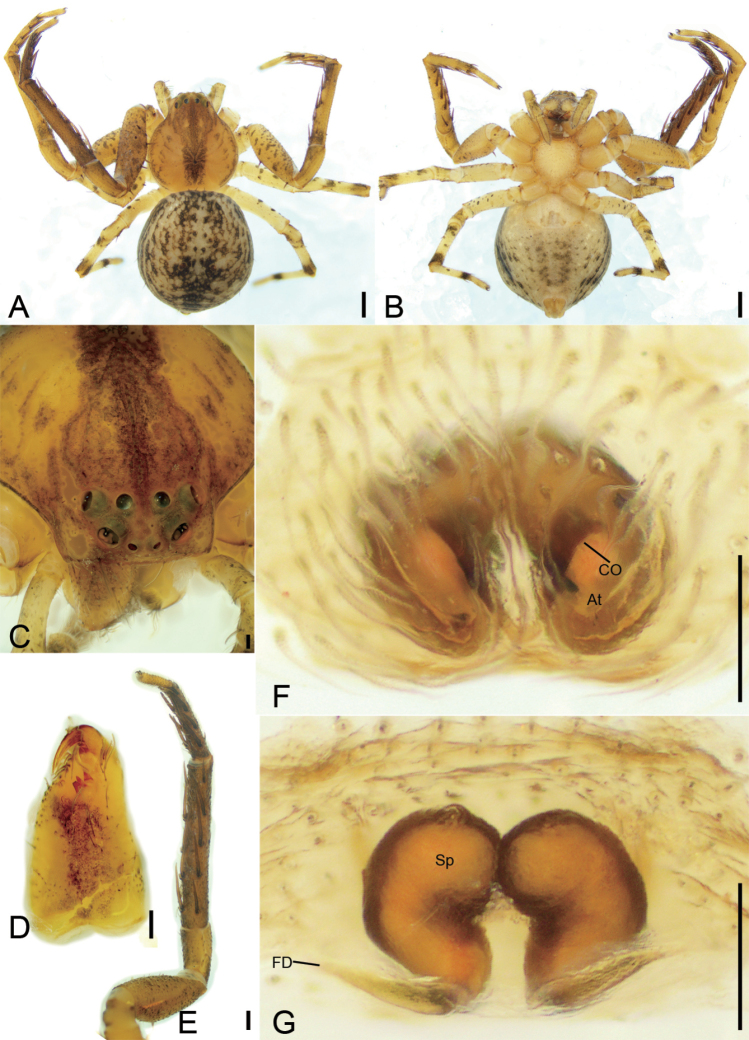
*Phartaxizang* sp. nov., female holotype, preserved **A** habitus, dorsal view **B** same, ventral view **C** eyes, dorsal view **D** chelicera, ventrolateral view **E** leg I, ventro-retrolateral view **F** epigyne, dorsal view **G** same, ventral view. Abbreviations: At – atrium, CO – copulatory opening, FD – fertilisation duct, Sp – spermatheca. Scale bars: 0.5 mm (**A, B**); 0.2 mm (**C**); 0.1 mm (**D–G**).

The male of this new species resembles *Phartatengchong* (Tang, Griswold & Yin, 2009) (see Tang et al. 2009: 47, fig. 6A−F) in having the prosoma with an inverted triangular black-brown marking and the touching anterior spermathecae with a bent part, but differs from it by the copulatory openings being hidden by cambered atrial lateral margins directed medially (vs. bilaterally in *P.tengchong*) and the separated posterior spermathecae (vs. closely touching in *P.tengchong*) (Fig. 4F, G).

#### Description.

**Female. *Habitus*** as in Fig. 4A, B. Total length 4.62, prosoma length 1.98, width 1.89, anteriorly narrowed to 0.50× its maximum width. Eye diameters (Fig. 4C): AME 0.05, ALE 0.13, PME 0.10, PLE 0.11; interdistances: AME−AME 0.10, AME−ALE 0.07, PME−PME 0.13, PME−PLE 0.14, AME−PME 0.19, AME−PLE 0.32, ALE−ALE 0.32, PLE−PLE 0.59, ALE−PLE 0.14. MOA 0.32 long, front width 0.20, back width 0.31. Chelicerae (Fig. 4D) with three small promarginal teeth and three retromarginal teeth (median and distal touching). Endites (Fig. 4B) nearly quadrilateral, slightly longer than wide. Labium (Fig. 4B) rectangular, wider than long, anteriorly with strong setae. Sternum (Fig. 4B) oval, longer than wide, with notch anteromedially. Legs measurements (Fig. 4A, B, E): I 6.12 (1.73, 0.78, 2.21, 1.04, 0.36); II 7.21 (2.27, 0.86, 2.09, 1.4, 0.59); III 3.19 (0.88, 0.4, 1.02, 0.45, 0.44); IV 4.67 (1.51, 0.58, 1.17, 0.91, 0.5); spination (Fig. 4A, B, E): I Fe: d5, p2, r1; Ti: p2, r2, v10; Mt: p1, r1, v8; II Fe: d2; Pa: p1; Ti: p3, r3, v10; Mt: p1, r1, v8; III Fe: d2; Pa: p1; Ti: d2, v2; Mt: d5, v1; IV: Fe: d1; Pa: d1; Ti: d2, p2, v1; Mt: d1, p2. Opisthosoma (Fig. 4A, B) length 2.63, width 2.40.

***Colouration*** (Fig. 4A, B). Prosoma yellow-brown, medially with single broad, dark brown, mottled band, laterally with fringe-shaped dark brown, mottled stripe. Chelicerae yellowish to dark brown. Endites yellow. Labium yellow-brown. Sternum yellow. Legs: tibia and metatarsus I yellow-brown, other segments yellow with a few dark spots, distal parts of tibiae and metatarsi III and IV with dark brown annulations. Opisthosoma grey to dark, with net-like mottled markings and sparse white guanine spots; venter yellow to dark brown, laterally with sparse white guanine spots.

***Epigyne*** (Fig. 4F, G). Epigyne heart-shaped, 1.2× wider than long. Copulatory openings (*CO*) visible, hidden by hood-shaped atrium (*At*). Copulatory ducts not visible, possibly absent. Spermathecae (*Sp*) C-shaped, ca. 2× longer than wide, anterior part of spermathecae closely touching, posterior parts slightly separated. Fertilisation ducts (*FD*) slightly less than the length of spermatheca, directed laterally.

#### Distribution.

Known only from the type locality in Tibet, China (Fig. 10).

### ﻿Genus Stephanopis

Taxon classificationAnimaliaAraneaeThomisidae

O. Pickard-Cambridge, 1869

025050B5-57D5-551B-8152-5AE973758B17

#### Comments.

This genus includes 24 species mainly distributed in Australasia, South America, and Asian mainland (WSC 2022). Nearly half of them (11 species) are described based either on single females or males (WSC 2022). Only one species was recorded from China on the Asian mainland, *S.xiangzhouica* Liu, 2022 (Liu et al. 2022c). Unfortunately, it is known only from the female in Jiangxi Province, China.

### ﻿ Stephanopis qiong

Taxon classificationAnimaliaAraneaeThomisidae

Liu & Yao sp. nov.

DB77FA27-68C1-5B67-906B-48D95AFCEF52

https://zoobank.org/3DC97292-727A-461B-84DA-AEB83E2AB902

Figs 5 , 6

#### Type material.

***Holotype*** ♀: China, Hainan, Ledong County, Jianfengling National Natural Reserve, Mingfenggu Scenic Spot, 18°44'25.87"N, 108°50'47.83"E, 4 April 2021, Yunhu Mo leg. (Tho-292, ASM-JGSU).

#### Etymology.

The specific name refers to the Chinese abbreviation for Hainan Province; noun in apposition.

#### Diagnosis.

**Figure 5.**
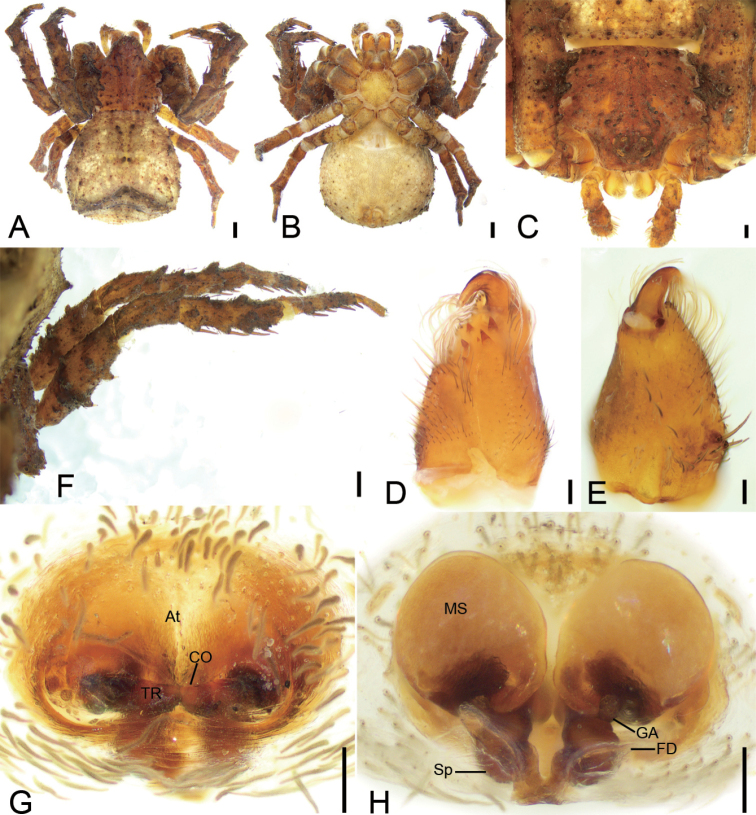
*Stephanopisqiong* sp. nov., female holotype, preserved **A** habitus, dorsal view **B** same, ventral view **C** eyes, dorsal view **D** chelicera, ventrolateral view **E** same, dorsolateral view **F** leg I, dorso-prolateral view **G** epigyne, dorsal view **H** same, ventral view. Abbreviations: At – atrium, CO – copulatory opening, FD – fertilisation duct, GA – glandular appendage, MS – membranous sac, Sp – spermatheca, TR – transverse ridge of copulatory openings. Scale bars: 0.5 mm (**A, B**); 0.2 mm (**C**); 0.1 mm (**D–H**).

The female of this new species resembles *Stephanopisxiangzhouica* Liu, 2022 (see Liu et al. 2022c: 64, fig. A, B) in having the copulatory openings hidden by a transverse ridge, but can be distinguished by the inverted heart-shaped atrium (vs. oval) and the tube-shaped spermathecae separated by nearly as long as their width (vs. the oval spermathecae separated by their half width) (Fig. 5G, H).

#### Description.

**Figure 6.**
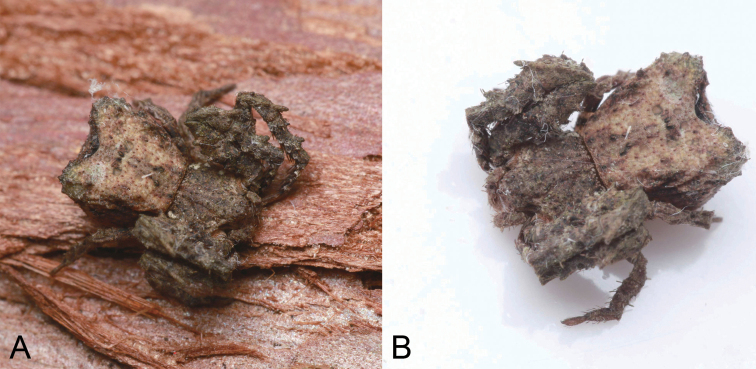
Photographs of living specimens from China **A, B***Stephanopisqiong* sp. nov., female.

**Female** (holotype). ***Habitus*** as in Figs 5A, B, 6C, D. Total length 5.46, prosoma (Fig. 5A, B) length 2.32, width 2.86, anteriorly narrowed to 0.43× its maximum width, covered with numerous strong, short, radially distributed peg-like setae and dense short plumose setae, with three rows of short, strong setae along the midline. Eye diameters (Fig. 5C): AME 0.05, ALE 0.11, PME 0.08, PLE 0.09; interdistances: AME-AME, 0.10, AME-ALE, 0.12, PME−PME, 0.25, PME−PLE, 0.14, AME−PME, 0.36, AME−PLE, 0.39, ALE−ALE, 0.37, PLE−PLE 0.67, ALE−PLE, 0.16. MOA 0.44 long, front width 0.19, back width 0.41. Chelicerae (Fig. 5D, E) with three promarginal and two retromarginal teeth, and a small denticle in-between. Endites (Fig. 5B) nearly quadrilateral, longer than wide. Labium (Fig. 5B) rectangular, wider than long, anteriorly with strong setae. Sternum oval, anteriorly flattened, as long as wide, covered by very dense setae. Legs measurements (Fig. 5A, B, F): I 9.17 (3.09, 1.53, 2.22, 1.55, 0.78); II 6.86 (1.97, 1.14, 2.03, 1.28, 0.44); III 4.67 (1.39, 0.75, 1.31, 0.67, 0.55); IV 5.85 (1.69, 0.73, 1.21, 1.73, 0.49); spination (Fig. 5A, B, F): I Ti: d1, v8; Mt: d1, r1, v8; II Ti: d2, v8; Mt: d2, v8; III Pa: d1; cusps: I Fe: 5; Pa: 2; Ti: 3; II Fe: 9; Pa: 3; Ti: 3; IV Fe: 2. Opisthosoma (Fig. 5A, B) length 3.14, width 3.87, pentagonal with a notch posteromedially; dorsum covered with sparse peg-like setae; venter with numerous plumose setae medially.

***Colouration*** (Fig. 5A, B). Prosoma reddish brown, with radial, irregular, dark brown mottled markings in the surface. Chelicerae, endites, labium, and sternum yellow-brown. Legs yellow to yellow-brown. Opisthosoma white to yellow, with numerous irregular guanine spots; venter yellow, with a few guanine spots on lateral parts.

***Epigyne*** (Fig. 5G, H). Epigyne oval, wider than long. Atrium (*At*) large, inverted heart-shaped, covering 2/3 of epigynal field. Copulatory openings (*CO*) invisible, hidden by a transverse ridge (*TR*). Copulatory ducts very short, touching. Membranous sacs (*MS*) transparent, located anteriorly, covering 2/3 of epigynal plate, slightly separated. Glandular appendages (*GA*) nearly spherical, almost as long as 1/2 width of spermatheca. Spermathecae (*Sp*) tube-shaped, slightly separated by less spermathecal width. Fertilisation ducts (*FD*) slightly less than the length of spermatheca, directed anterolaterally.

**Male.** Unknown.

#### Distribution.

Known only from the type locality in Hainan Province, China (Fig. 10).

### ﻿ Stephanopis xiangzhouica

Taxon classificationAnimaliaAraneaeThomisidae

Liu, 2022

B839BFBA-4068-53AB-9F7C-375A8A5938CE

Figs 7 , 8 , 9

Stephanopis xiangzhouica Liu, in Liu et al. 2022c: 64, figs 12A−I, 13A, B (holotype ♀ from Jinggang National Nature Reserve, Jiangxi Province, deposited in ASM-JGSU, No. Tho-17, examined).

#### Material examined.

1 ♂, 1 ♀, China, Guangdong, Ruyuan County, Nanling National Natural Reserve, Waterfalls Scenic Spot, 24°54'52.11"N, 113°2'28.67"E, 779 m, 6 September 2020, Qingbo Huo leg. (Tho-295, ASM-JGSU).

#### Diagnosis.

**Figure 7.**
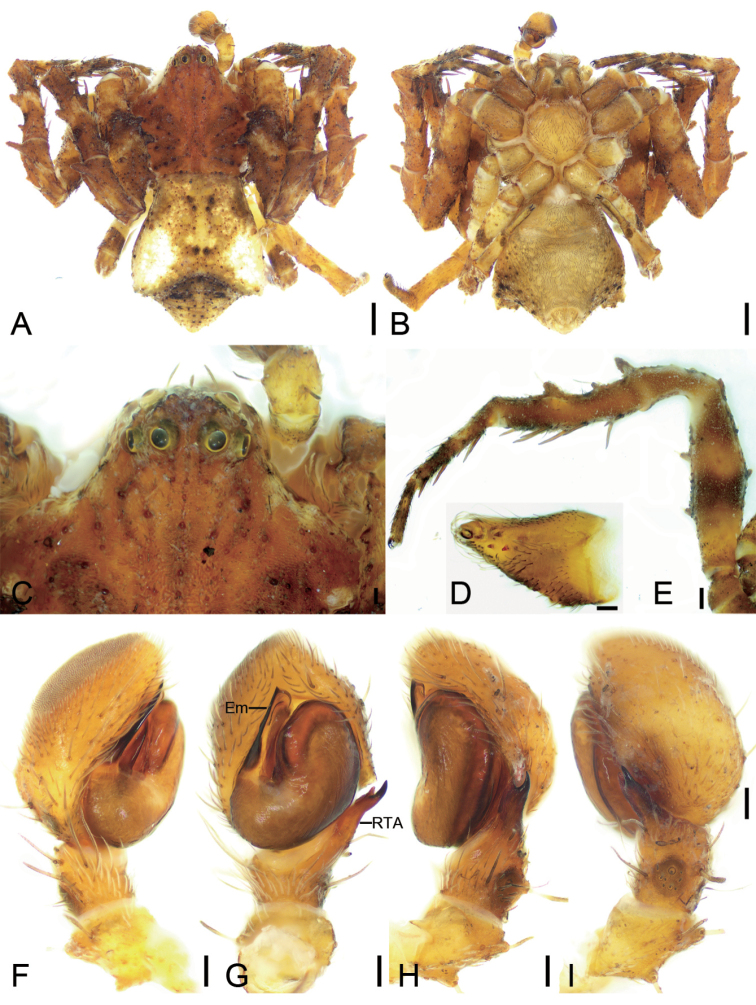
*Stephanopisxiangzhouica* Liu, 2022, male, preserved **A** habitus, dorsal view **B** same, ventral view **C** eyes, dorsal view **D** chelicera, ventral view **E** leg I, retrolateral view **F** palp, prolateral view **G** same, ventral view **H** same, ventro-retrolateral view **I** same, retro-dorsal view. Abbreviations: Em – embolus, RTA – retrolateral tibial apophysis. Scale bars: 0.5 mm (**A, B**); 0.2 mm (**C**); 0.1 mm (**D–I**).

**Figure 8.**
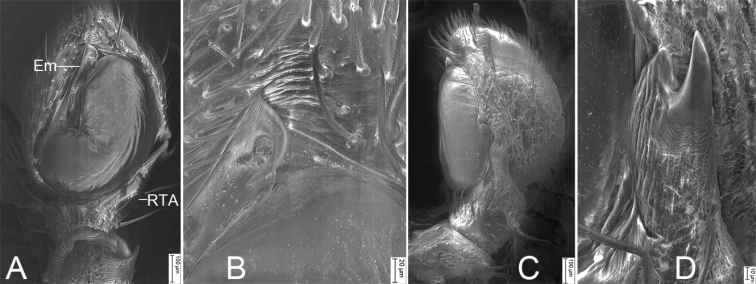
SEM micrographs of *Stephanopisxiangzhouica* Liu, 2022, male palp **A** ventral view **B** same, details of embolus **C** retrolateral view **D** same, detail of retrolateral tibial apophysis. Abbreviations: Em – embolus, RTA – retrolateral tibial apophysis.

The male of this species resembles *S.nigra* O. Pickard-Cambridge, 1869 (see Machado et al. 2019: fig. 38C, D) in having a forked retrolateral tibial apophysis, but it can be distinguished by the retrolateral tibial apophysis being longer than tibia (vs. less than tibial length) and the embolus with a hook-shaped apex (vs. flagelliform) (Figs 7F−I, 8). The male of this species also resembles *S.altifrons* O. Pickard-Cambridge, 1869 (see Machado et al. 2019: 224, fig. 3C−F), *S.carcinoides* Machado, 2019 (see Machado et al. 2019: 243, fig. 20C, D), and *S.lata* O. Pickard-Cambridge, 1869 (see Machado et al. 2019: 253, fig. 29C, D), but it can be easily distinguished from them by the embolus having a hook-shaped apex (vs. flagelliform in all three species) and the retrolateral tibial apophysis with two morphologically different branches (dorsal branch much longer and thicker than the ventral) (vs. ventral branch much longer and thicker than the dorsal in *S.altifrons* and *S.carcinoides*; ventral branch indistinct in *S.lata*) (Figs 7F−I, 8). Female diagnosis as in Liu et al. (2022c).

#### Description.

**Male. *Habitus*** as in Fig. 7A, B. Total length 4.77, prosoma length 2.17, width 2.29, anteriorly narrowed to 0.37× its maximum width, covered with numerous strong, short, radially peg-like setae and dense short plumose setae, with three rows of short strong setae along midline. Eye diameters (Fig. 7C): AME 0.06, ALE 0.13, PME 0.10, PLE 0.10; interdistances: AME−AME 0.09, AME−ALE 0.05, PME−PME 0.21, PME−PLE 0.09, AME−PME 0.28, AME−PLE 0.34, ALE−ALE 0.13, PLE−PLE 0.60, ALE−PLE 0.13. MOA 0.42 long, front width 0.22, back width 0.41. Chelicerae (Fig. 7D) three promarginal teeth and one retromarginal tooth. Endites (Fig. 7B) nearly quadrilateral, longer than wide, laterally with long setae. Labium (Fig. 7B) rectangular, wider than long, anteriorly with strong setae. Sternum round, nearly as long as wide, covered by dense setae. Legs measurements (Fig. 7A, B, E): I 4.49 (1.62, 0.73, 1.12, 0.67, 0.35); II 4 (1.35, 0.73, 0.98, 0.6, 0.34); III 4.4 (1.37, 0.71, 1.08, 0.67, 0.57); IV 4.6 (1.63, 0.65, 1.06, 0.68, 0.58); spination (Fig. 7A, B, E): I Fe: v2; Ti: d2, v8; Mt: v8; II Fe: v4; Ti: v8; Mt: d3, v8; III Ti: p1; cusps: I Fe: 11; Pa: 4; Ti: 5; Mt: 2; II Fe: 11; Pa: 4; Ti: 4. Opisthosoma (Fig. 7A, B) length 2.58, width 2.11, pentagonal with pair of latero-posterior horns; dorsum covered with sparse brown peg-like and small, dense, plumose setae; venter with numerous plumose setae.

***Colouration*** (Fig. 7A, B). Prosoma reddish brown. Chelicerae, endites, and labium yellow-brown. Sternum yellow, with yellow-brown margin. Legs mottled, I and II yellow to reddish brown, III and IV grey to yellow. Opisthosoma grey to yellow-brown, laterally with numerous irregular guanine spots; venter yellow.

***Palp*** (Figs 7F−I, 8). Palp with a long retrolateral tibial apophysis (*RTA*), pincer-like in retrolateral view, longer than tibia; embolus (*Em*) flatted-shaped, with broad base, less than tegular length, originating at approximately the 8 o’clock position of the tegulum, with a distinct constriction in the subapical part, and a hook-shaped apex.

**Figure 9.**
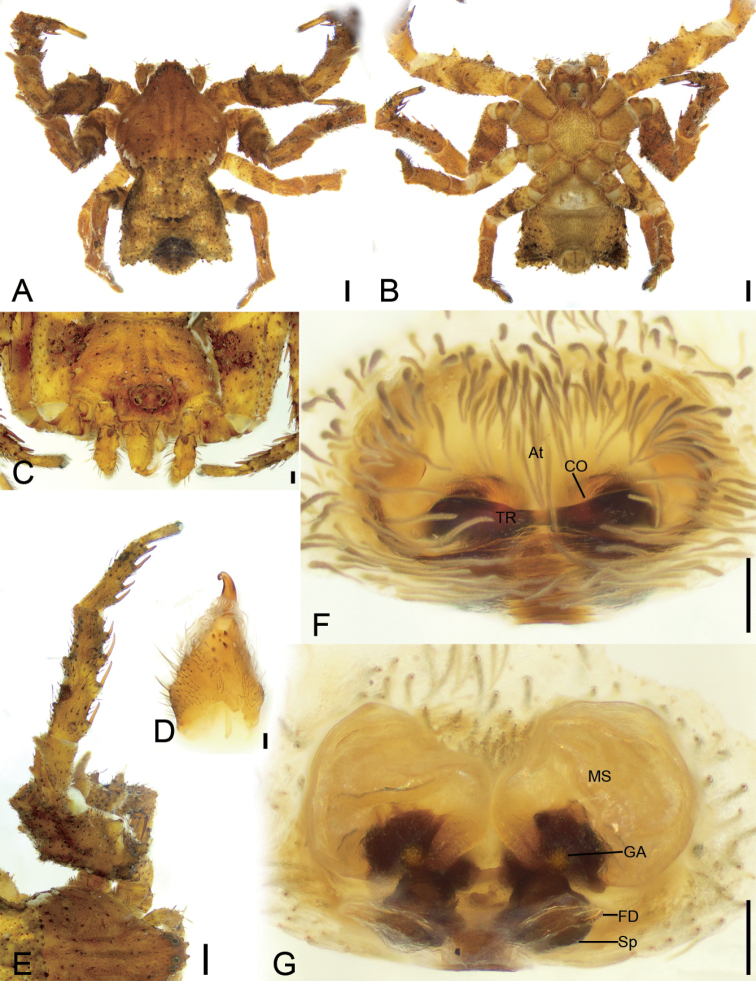
*Stephanopisxiangzhouica* Liu, 2022, female, preserved **A** habitus, dorsal view **B** same, ventral view **C** eyes, dorsal view **D** chelicera, ventral view **E** leg I, prolateral view **F** epigyne, dorsal view **G** same, ventral view. Abbreviations: At – atrium, CO – copulatory opening, FD – fertilisation duct, GA – glandular appendage, MS – membranous sac, Sp – spermatheca, TR – transverse ridge of copulatory openings. Scale bars: 0.5 mm (**A, B**); 0.2 mm (**C**); 0.1 mm (**D–G**).

**Female.** Description in Liu et al. (2022c) for female sex. Female habitus shown in Fig. 9A, B; eyes, chelicerae, and leg I in Fig. 9C−E; and epigyne in Fig. 9F, G.

#### Distribution.

**Figure 10.**
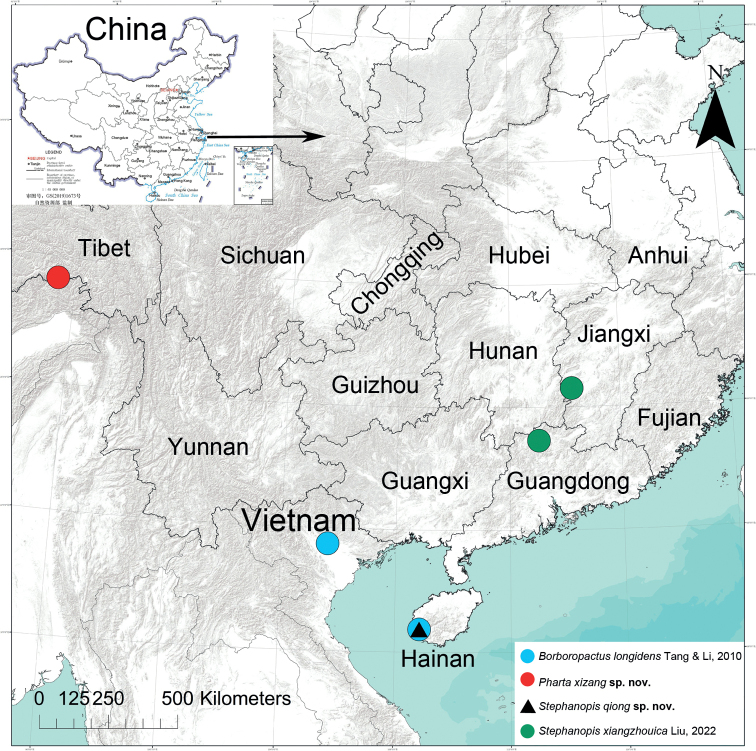
Records of *Borboropactuslongidens* Tang & Li, 2010, *Phartaxizang* sp. nov., *Stephanopisqiong* sp. nov. and *S.xiangzhouica* Liu, 2022 from Asia.

Known from Jiangxi and Guangdong provinces, China (Fig. 10).

## Supplementary Material

XML Treatment for Borboropactus

XML Treatment for Borboropactus longidens

XML Treatment for Pharta

XML Treatment for Pharta xizang

XML Treatment for Stephanopis

XML Treatment for Stephanopis qiong

XML Treatment for Stephanopis xiangzhouica
